# Texture feature analysis of MRI-ADC images to differentiate glioma grades using machine learning techniques

**DOI:** 10.1038/s41598-023-41353-5

**Published:** 2023-09-22

**Authors:** Sahan M. Vijithananda, Mohan L. Jayatilake, Teresa C. Gonçalves, Luis M. Rato, Bimali S. Weerakoon, Tharindu D. Kalupahana, Anil D. Silva, Karuna Dissanayake, P. B. Hewavithana

**Affiliations:** 1 Department of Radiology, Faculty of Medicine, University of Peradeniya, Peradeniya, 20400 Sri Lanka; 2 Department of Radiography/Radiotherapy, Faculty of Allied Health Sciences, University of Peradeniya, Peradeniya, 20400 Sri Lanka; 3 Department of Informatics, University of Évora, 7000 Évora, Portugal; 4 Department of Computer Engineering, Faculty of Engineering, University of Sri Jayawardhanapura, Dehiwala-Mount Lavinia, Sri Lanka; 5 Department of Radiology, National Hospital of Sri Lanka, Colombo 10, 01000 Sri Lanka; 6 Department of Histopathology, National Hospital of Sri Lanka, Colombo 10, 01000 Sri Lanka

**Keywords:** Cancer, Computational biology and bioinformatics, Neurology, Engineering

## Abstract

Apparent diffusion coefficient (ADC) of magnetic resonance imaging (MRI) is an indispensable imaging technique in clinical neuroimaging that quantitatively assesses the diffusivity of water molecules within tissues using diffusion-weighted imaging (DWI). This study focuses on developing a robust machine learning (ML) model to predict the aggressiveness of gliomas according to World Health Organization (WHO) grading by analyzing patients’ demographics, higher-order moments, and grey level co-occurrence matrix (GLCM) texture features of ADC. A population of 722 labeled MRI-ADC brain image slices from 88 human subjects was selected, where gliomas are labeled as glioblastoma multiforme (WHO-IV), high-grade glioma (WHO-III), and low-grade glioma (WHO I-II). Images were acquired using 3T-MR systems and a region of interest (ROI) was delineated manually over tumor areas. Skewness, kurtosis, and statistical texture features of GLCM (mean, variance, energy, entropy, contrast, homogeneity, correlation, prominence, and shade) were calculated using ADC values within ROI. The ANOVA f-test was utilized to select the best features to train an ML model. The data set was split into training (70%) and testing (30%) sets. The train set was fed into several ML algorithms and selected most promising ML algorithm using K-fold cross-validation. The hyper-parameters of the selected algorithm were optimized using random grid search technique. Finally, the performance of the developed model was assessed by calculating accuracy, precision, recall, and F1 values reported for the test set. According to the ANOVA f-test, three attributes; patient gender (1.48), GLCM energy (9.48), and correlation (13.86) that performed minimum scores were excluded from the dataset. Among the tested algorithms, the random forest classifier(0.8772 ± 0.0237) performed the highest mean-cross-validation score and selected to build the ML model which was able to predict tumor categories with an accuracy of 88.14% over the test set. The study concludes that the developed ML model using the above features except for patient gender, GLCM energy, and correlation, has high prediction accuracy in glioma grading. Therefore, the outcomes of this study enable to development of advanced tumor classification applications that assist in the decision-making process in a real-time clinical environment.

## Introduction

Glioma is the most common primary neoplasm type in the central nervous system (CNS). According to the epidemiology of intracranial neoplasms, 30% of all the primary CNS neoplasms, as well as 80% of intracranial malignancies that occur in adults, are gliomas^1–3^. However, gliomas can be defined as an abnormal and uncontrollable proliferation of glial cells/neuroglia by bypassing the mechanisms that control the normal cell division to form a heterogeneous group of neoplastic masses belonging to multiple histologic types and malignancy grades^4,5^. Glial cells refer to all the non-neuronal cells that are associated with both CNS and the peripheral nervous system (PNS). The ependymal, oligodendrocytes, astrocytes, and choroid plexus cells are identified as the glial cells that are responsible to maintain the structural integrity of the CNS and PNS while providing metabolic support; nutrient and waste transportation, communication, and insulation to the adjacent neurons. Therefore, the term glioma is considered as a non-specific term that indicates the origin of the tumor is from one of the types of glial cells; i.e., ependymoma, oligodendroglioma, astrocytoma, and choroid plexus papilloma arises from the ependymal, oligodendrocytes, astrocytes, and choroid plexus cells respectively^6^. According to the World Health Organization (WHO), gliomas are classified into four grades (I, II, III, and IV) by considering the aggressiveness (histological and molecular features) of the tumors^7–9^. However, medical imaging including Magnetic Resonance Imaging (MRI), Computed Tomography (CT), Positron Emission Tomography and Computed Tomography (PET-CT), PET-Magnetic Resonance and other nuclear imaging modalities such as scintigraphy plays a major role in brain tumor diagnosis, identification, and therapeutic procedures^10–12^.

### Magnetic resonance imaging

Among the above-mentioned medical imaging modalities, MRI is one of the most promising neuroimaging modalities that are being used in the current clinical setup to produce diagnostic medical images of brain tumors^13^. There are a number of MRI sequences; T1 weighted, T2 weighted, Fluid Attenuation and Inversion Recovery (FLAIR), Diffusion-Weighted Imaging (DWI), T1 post-contrast fast-spin echo (T1 FSE), and susceptibility-weighted imaging (SWI) are currently being used in routine neuroimaging practices. Among the above sequences, the DWI images have the ability to probe the random Brownian motions of water molecules within tissues on a voxel basis^6,14^. As the DW images provide information about the net direction of the water molecules within tissues, it is widely appreciated in observing the microscopic behavior of biological tissues; the existence of membranes, cellularity, the intracellular-extracellular water equilibrium, and the presence of macromolecules. Changes in the microscopic diffusion of water molecules within tissue indicate the alteration of homeostasis at the cellular level^15^. Therefore, DWI became an indispensable tool in clinical neuroimaging. It became widely popular among clinicians as a powerful imaging tool for the diagnosis of some life-threatening conditions such as ischemic, tumors, trauma, and non-life-threatening conditions like schizophrenia, multiple sclerosis, dyslexia, etc^16–19^.

### Diffusion weighted imaging and apparent diffusion coefficient

DWI can be acquired at different diffusion sensitization levels by changing the critical parameters; the amplitude and duration of the applied diffusion sensitization gradient. These parameters have the ability to encode different properties of tissues into DWI signals while controlling the magnitude of diffusion weighting in the resultant image. The sensitivity of the acquired DW image is indicated by the *b-value* that is measured in seconds per square millimeter (s/mm$^2$). The *b-value* is also proportional to the duration, and square of the amplitude of the applied diffusion sensitization gradient. The diffusion of water molecules within tissues is qualitatively assessed by trace DW images and it is being quantitatively assessed by calculating the apparent diffusion coefficient (ADC) parameter (see Eq.  1). According to Eq. (1), it is mandatory to have the involvement of at least two DW images with different *b-values* to calculate ADC values. The images with *b* = 0 s/mm$^2$ are utilized as the lower limit in common radiology practices while *b-values* from 600 to 1000 s/mm$^2$ are used for the upper limit^20,21^. However, *b-values* greater than 1000 s/mm$^2$ are also applied to generate ADC in non-routine studies^22^. The degree of the diffusion of water molecules through adjacent structures is visualized by plotting the calculated ADC values as a parametric map. High ADC values represent less impedance for the diffusion of water molecules within tissues, and such tissues are hyperintense in ADC while hypointense in trace DW images. As a result, these hyperintense and hypo-intensities express different textures for different tissue types according to their microscopic behavior.

MRI-ADC imaging provides information about tissue microstructure by assessing water diffusion. It detects changes in cellular density and organization, which are indicative of diseases. Lower ADC values suggest higher cellular density and disrupted tissue architecture, highlighting pathological alterations. MRI-ADC imaging is sensitive to early microstructural changes, enabling early disease diagnosis. It offers diagnostic value in oncology, neurology, and other medical fields. Being non-invasive, it provides valuable insights without the need for invasive procedures.

### Image texture

Texture describes the structure and surface of an image by considering the regular repetition of an element or pattern on the surface. Image texture provides important information about the spatial arrangement of intensities or colors in an image^23,24^. The ADC images have the ability to visualize the structures with different diffusivity in different grey levels/different intensities which make the image enriched with texture. Texture analysis is based on finding the specific patterns of hidden characteristics of the texture and presenting them in a more simplified and unique way. Grey Level Co-occurrence Matrix (GLCM) can be identified as a promising statistical method to examine the texture of an image by considering the spatial relationship of pixels^25^. GLCM is a square matrix with dimensions equal to the number of grey levels (n $\times$ n) contained in the 2D parametric ADC image (*I*) and it counts the co-occurrence of neighboring grey levels of pixels within the image along $0^\circ , 45^\circ , 90^\circ$, and $135^\circ$ orientations and summed^26,27^.

### Higher order moments

Apart from the first and second-order statistics such as mean, and variance, the higher-order statistics (HOS) have also played a tremendous role in signal processing and system analysis in recent history. The higher-order statistics are the statistical functions that use high power of sample; higher than 2nd order (lower order) statistics, provide useful tools for addressing issues in nonlinear systems^28^. Higher-order statistics such as third-order (Skewness), and fourth-order (Kurtosis) carry more useful information due to their phase sensitiveness. Such information is critical in developing robust statistical modes to identify non-minimum phase systems^29,30^. In third-order statistics; skewness measures the asymmetry around the mean of a probability distribution of a data set. The skewness of a normal distribution remains at zero. However, the distributions skewed to left are indicated by negative (−) values while the distributions skewed to right are indicating positive (+) values. The distributions with skewness value less than −0.5 or higher than 0.5 are considered highly skewed distributions. Kurtosis measure and compare the shape (tail) of the probability distribution of a real-valued random variable with a normal distribution. The kurtosis value of any univariate normal distributions remains at 3 and the distributions with kurtosis of more than 3 are considered as platykurtic distributions. In contrast, the distributions with kurtosis of less than 3 are identified as leptokurtic distributions^31^.

### Machine learning

Machine learning is a branch of artificial intelligence (AI) that allows computers to “learn” from data and develop analytical models to aid and/or support in making decisions and predictions with minimal human involvement. Here, the ML algorithms are used to identify the hidden characteristics/patterns of data to develop analytical models. ML approaches can be classified into three main categories: Supervised learning, Unsupervised learning, and Reinforcement learning^32^. Supervised learning uses labeled datasets to train ML algorithms while unsupervised learning uses unlabeled datasets to train. Reinforcement learning is a type of machine learning that learns as it goes by using trial and error. Supervised learning is a powerful learning method that is being used to address a variety of real-world classification, and regression problems^33,34^.

**Figure 1:**
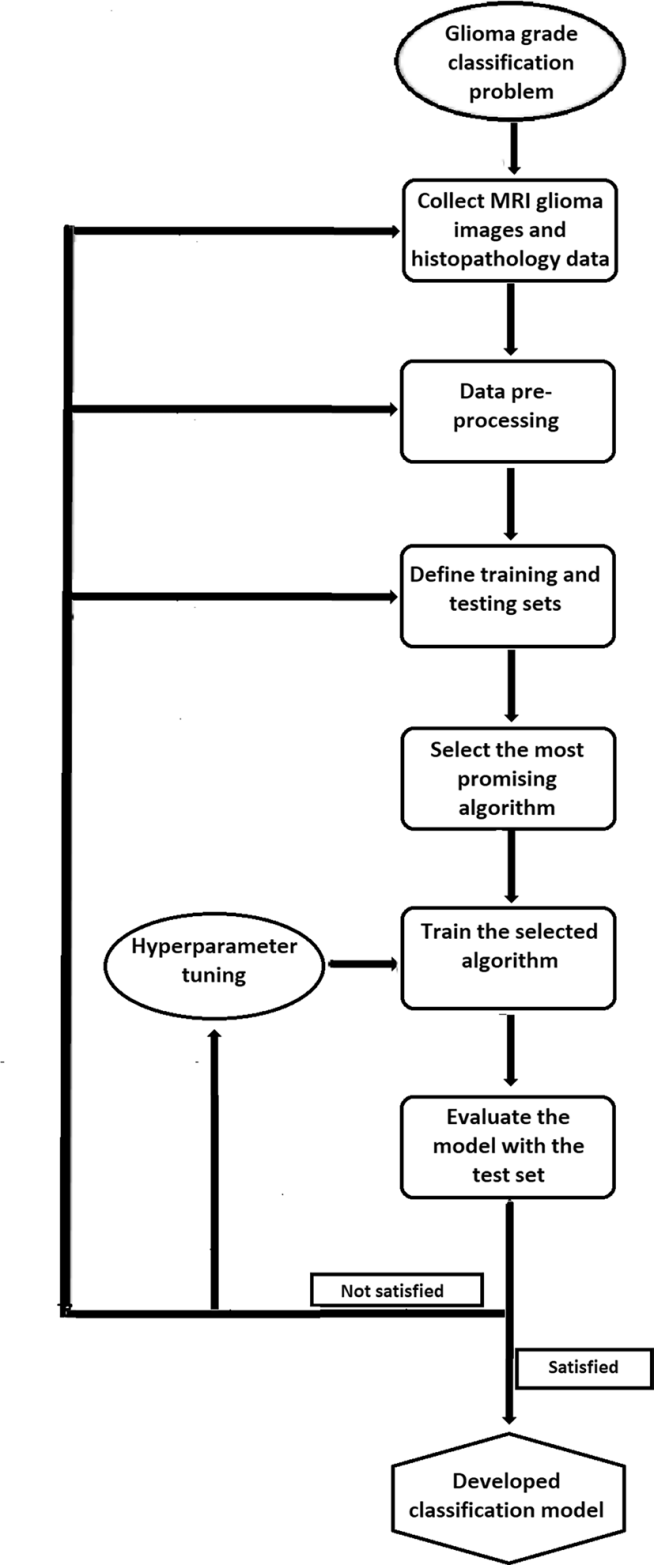
Application of supervised learning method for multiclass tumor classification problem. The flow chart illustrates the steps followed in developing a multiclass classification model. After identification of the nature of the problem, the necessary MRI and histopathology data are collected. At the data pre-processing step, the texture features are extracted from MRI images, and the extracted data is prepared to be compatible with training the machine learning model (data labeling, removing defected data, binarization). The next step splits the dataset into train and test sets. The most promising machine learning algorithm for the dataset is selected and fed to the algorithm with the training set to build the classification model. Finally, the performance of the developed model is assessed. When the performance did not meet the required level of performance, the hyperparameters of the developed model are tuned and find the most suitable combination of hyperparameters. Also, sometimes it is necessary to revise the data collection, data pre-processing, and repeat training and testing steps until meeting the required performance of the model.

Therefore, the supervised learning method can be identified as one of the most common ML paradigms that use labeled input data to train ML algorithms^25,35–37^. When the data is fed into the algorithm, it identifies the hidden characteristics, patterns, and correlations for each class and makes ML models using such information. The process iterates until the algorithm achieves the highest prediction accuracy and the developed model is able to address the intended problem with high accuracy level (see Fig. 1). The accuracy level of the developed ML model is optimized by tuning the hyper-parameters of the model^38^. Among the various types of supervised learning algorithms, Neural Networks, Naïve Bayes, Linear Regression, Logistic Regression, Support Vector Machines, K-Nearest Neighbor, Decision Tree, and Random Forest algorithms are the most commonly used ones. From the above algorithms, the Random Forest algorithm can be identified as an ensemble method that uses a collection of decision trees to generate a decision in classification and regression problems.

### Literature survey

There are numerous studies available in the literature that focus on the development of glioma grade classification models. In recent years, several notable studies have contributed to this field. When we consider few of most recent resent studies, in year 2019, A. Vamvakas et al., developed support vector machine (SVM) binary classification model to predict glioma types (High grade glioma, Low grade glioma) using the radiomics features extracted from several MRI image sequences including T1 pre/post-contrast, T2-FSE, T2-FLAIR (Fluid Attenuation and Inversion Recovery) Diffusion Tensor, Perfusion Imaging and 1H-MR Spectroscopy. As a result, they could predict these two classes of gliomas with 95.5% Accuracy^39^. Another study conducted in 2019 by Nidhi Gupta et al. involved the development of a model to identify and classify gliomas using MRI images in T1, T1-post contrast, T2, and FLAIR sequences. The researchers incorporated image texture features, as well as morphological and inherent characteristics of the tumor such as solidity, perimeter, area, and orientation. Their classification model achieved an accuracy of 97.76%^40^.

In another study conducted in 2017 by Xin Zhang et al., various machine learning methods were compared for glioma grading, specifically distinguishing between low grade and high grade gliomas, using multi-parametric MRI data. The study extracted quantitative parameters, including parametric histogram and image texture attributes, from perfusion, diffusion, and permeability maps of gliomas. The SVM method achieved a classification accuracy of 94.5% in differentiating between the two glioma classes^41^. In the year 2012, Nitish Zulpe1 and Vrushsen developed a brain tumor classification model using the GLCM texture features extracted from T2 weighted and proton density (PD) MRI image sequences obtained from four subjects with four different types of brain tumors. However, the model developed in a two-layered Feedforward Neural Network predicted the tumor types with 97.5% average accuracy^42^. Jiang et al., in the year 2017 developed a statistical model to discriminate low grade and high-grade gliomas by using the texture features extracted from multiple types of MRI sequences such as T2-FLAIR and T1WI-Contrast enhanced DWI sequences and found GLCM cluster shade, entropy and homogeneity as the best features to use in differentiating low grade and high grade gliomas^43^. Rajagopal et al., (2019) developed a glioma detection and segmentation model using GLCM features extracted from the MRI brain images and they utilized the random forest classifier to build the classification model with an accuracy of 97.7%^44^.

In the year 2019, Reza et al. proposed a high grade and low-grade glioma classification model developed in random forest classifier was able to classify gliomas with significantly high accuracy the model developed in SVM^45^. However, in the study, the texture features of MRI brain images have been acquired from multiple MRI sequences such as T1 weighted, T2 weighted, T1- post-contrast, and FLAIR.

In the year 2019, Deniz Alis et al., developed machine learning model to predict IDH1 status in high-grade gliomas. This study used texture features extracted from axial T2WI FLAIR, post-contrast T1WI, and ADC maps to feed random forest classifier. The developed model was able to predict IDH1 status of high-grade gliomas with 86.94% accuracy^46^. Similarly, Han et al. (2018) used ADC-based texture features along with other clinical and radiological features to classify gliomas into three different grades and achieved an accuracy of 89.6%^47^.

The study conducted by Radwa et al., in the year 2021 was able to find a significant difference of mean ADC values between high-grade glioma (HGG) and low-grade glioma (LGG) by analyzing the features extracted from ADC images of gliomas^48^. similarly, in the year 2018, Fusun et al. developed a machine learning model based on support vector machine to differentiate between high-grade glioma (WHO III and IV) and low-grade gliomas (WHO I and WHO II) using the features extracted from T1 and T2-weighted, diffusion-weighted, diffusion tensor, MR perfusion and MR spectroscopic imaging. However, their binary classification model was able to classify two glioma classes with an accuracy of 93.0%^49^. In summary, classifying glioma using MR images is a prevalent research problem among the scientific community focused on the advancement of medical imaging.

Almost all the studies discussed in the literature use at least T1-post-contrast images that involve invasive procedures. However, the literature currently lacks strong evidence for a method developed to differentiate glioma grades solely based on texture features extracted from MRI-ADC images and avoid any kind of invasive procedures. Here in this study, over aim was to address this gap in the literature and generate novel insights and contribute to the existing knowledge base in this field. The proposed non-invasive approaches aim to provide accurate classification results while minimizing patient discomfort and potential risks associated with contrast agents.

### Hypothesis

The study is based on the hypothesis; that there is an existence of a correlation between the extracted features (patients’ demographics, higher-order moments of ADC, and GLCM texture features of ADC) and the severity level of the glioma (WHO glioma grading levels).

### Objectives

Objectives of this study include the development of a robust and non-invasive method for distinguishing between low-grade glioma (WHO I/II), high-grade glioma (WHO III), and glioblastoma (WHO IV) based on features extracted from MRI-ADC images. This will be achieved through the analysis of patients’ demographics, higher-order moments of ADC, and GLCM texture features of ADC using machine learning techniques. The primary aim of the study is to improve the accuracy of glioma diagnosis, which will ultimately lead to better patient outcomes with zero invasive procedures and minimum patient discomfort. This research will contribute to the advancement of medical knowledge in the field of neuro-oncology and may have significant implications for clinical practice.

### Main contributions

The main contributions of this work are:

- Development of a robust and non-invasive method: The study aims to develop a robust and non-invasive method for distinguishing between low-grade glioma (WHO I/II), high-grade glioma (WHO III), and glioblastoma (WHO IV). This method will be achieved through the analysis of patients’ demographic information, higher-order moments of ADC, and GLCM texture features of ADC using machine learning techniques.
- Improvement of glioma diagnosis accuracy in a noninvasive manner: The primary aim of the study is to improve the accuracy of noninvasive glioma classification, which will ultimately lead to better patient outcomes with minimum patient discomfort.
- Advancement of medical knowledge in the field of neuro-oncology: This research will contribute to the advancement of medical knowledge in the field of neuro-oncology by providing new insights into the diagnosis and classification of gliomas. The study may also lead to the discovery of new biomarkers or imaging features that can be used to improve the diagnosis and treatment of gliomas.
- Potential implications for clinical practice: The findings of this study may have significant implications for clinical practice by providing clinicians with a more accurate and reliable method for diagnosing gliomas. This could lead to improved patient outcomes and a reduction in the number of unnecessary biopsies or surgeries.

## Results

**Figure 2:**
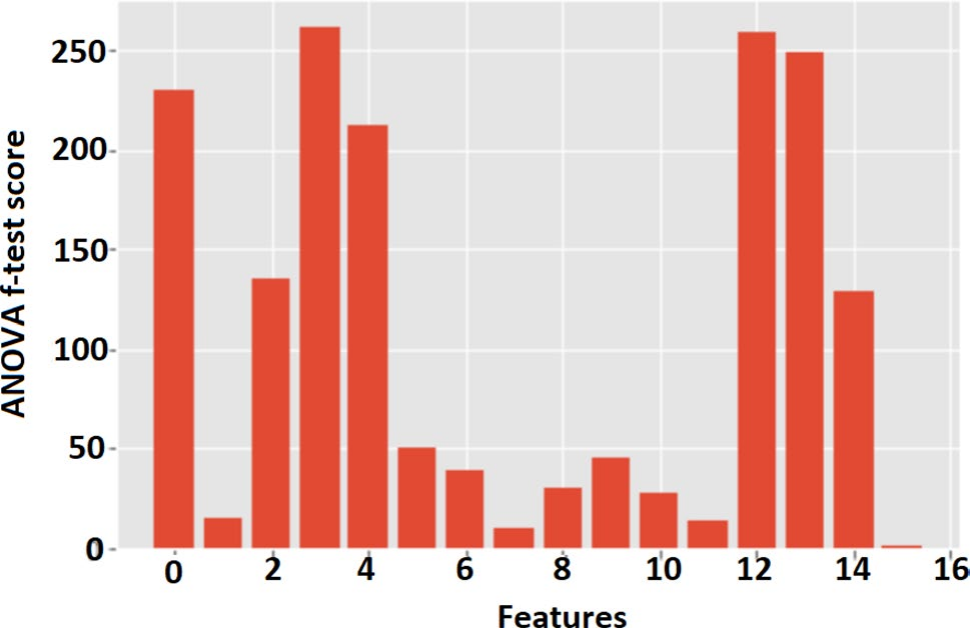
ANOVA F-test results in a bar chart. The figure illustrates the bar chart of the feature importance scores for each input feature. The standardized features; mean ADC, skewness, kurtosis, GLCM mean 1, GLCM mean 2, GLCM variance 1, GLCM variance 2, energy, entropy, contrast, homogeneity, correlation, prominence, shade patient’ age, and gender are indicated by 0 to 15 numbers in the bar chart respectively.

According to the results of the analysis of variance (ANOVA) F-test feature selection, the patient gender (1.4850), GLCM Energy (9.4805), and the GLCM Correlation (13.8695) were excluded from the dataset as such features reported the minimum scores (see Table 1) (see Fig. 2). Among the seven ML algorithms tested in the tenfold cross-validation process, the Random Forest Classifier reported the maximum mean-cross-validation score (mean-accuracy) for both balanced (0.8772 ± 0.0237) and imbalanced (0.7901 ± 0.0495) datasets. Therefore, the Random Forest Classifier was selected as the basic tool for building the glioma classification model (see Table 2).

**Table 1:** ANOVA F-test scores for each feature.

Feature	ANOVA F-test score
Mean ADC	230.8198
Skewness	14.3727
kurtosis	135.5591
GLCM mean 1	262.2695
GLCM mean 2	212.9521
GLCM variance 1	50.6215
GLCM variance 2	38.5333
Energy	9.4805
Entropy	30.3616
Contrast	44.8493
Homogeneity	27.6501
Correlation	13.8695
Prominence	258.8106
Shade	249.7605
Patients’ age	128.6092
Patients’ gender	1.4850

The table illustrates the performance of each feature at the ANOVA F-test feature selection process.

**Table 2:** The mean cross-validation scores, standard deviation (SD) and the accuracy from different algorithms for the balanced and imbalanced datasets.

	Algorithm	Mean accuracy	(SD)	Accuracy as percentage
With SMOTE	k-Nearest neighbors classifier	0.8398	0.0227	83.98%
	Linear discriminant analysis	0.7181	0.0385	71.81%
	Gaussian Naïve Bayes	0.6640	0.0420	66.40%
	Decision tree classifier	0.8176	0.0507	81.76%
	Support vector machine	0.8132	0.0407	81.32%
	Random forest classifier	0.8772	0.0237	87.72%
	Logistic regression	0.7457	0.0371	74.57%
Without SMOTE	k-Nearest neighbors classifier	0.6969	0.0541	69.69%
	Linear discriminant analysis	0.7167	0.0501	71.67%
	Gaussian Naïve Bayes	0.6927	0.0525	69.27%
	Decision tree classifier	0.7443	0.0877	74.43%
	Support vector machine	0.6772	0.0484	67.72%
	Random forest classifier	0.7901	0.0495	77.83%
	Logistic regression	0.6910	0.0457	69.10%

The table illustrates the mean K-fold cross-validation scores and the corresponding standard deviations acquired by each classification algorithm with and without the application of the synthetic minority over-sampling technique (SMOTE) over the dataset.

**Figure 3:**
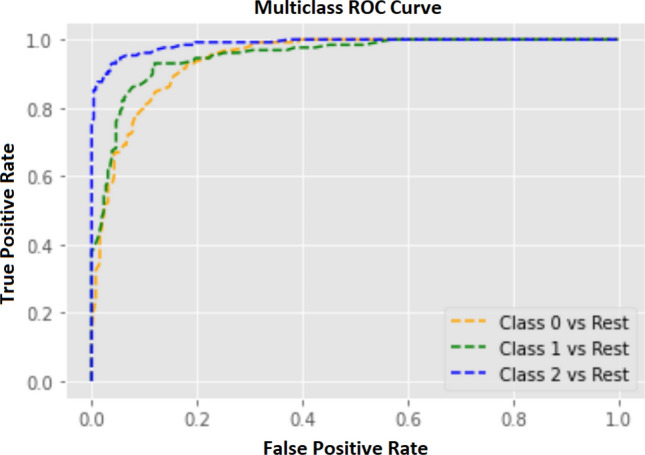
Multiclass receiver operating characteristic (ROC) curve for the base model. The ROC curve illustrates the trade-off between true positives and false positives that reflects the performance of the classification model at various threshold settings. The performance of multiclass classification models is displayed in ROC curve s using the one vs rest technique. Class 0, 1, and 2 represent glioblastoma, high-grade glioma, and low-grade glioma, respectively. The area under the curve (AUC) for each curve; yellow: 0.9434, green: 0.9521, and blue: 0.9885.

**Figure 4:**
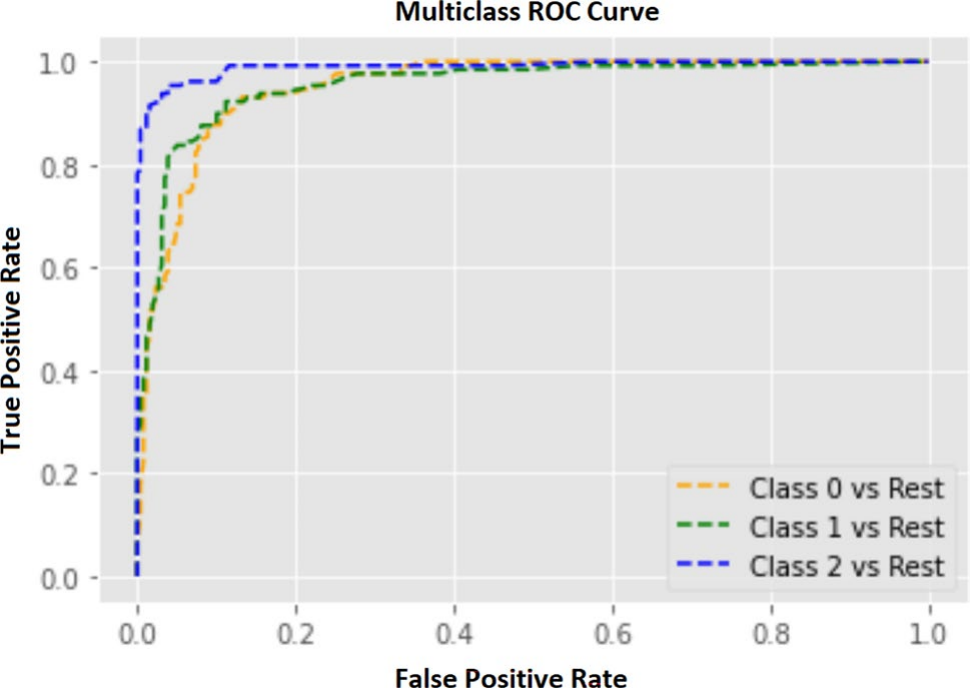
Multiclass receiver operating characteristic (ROC) curve for the base model after hyperparameter tuning. The performance of tuned multiclass classification models is displayed in ROC curves using the one vs rest technique. Class 0, 1, and 2 represent glioblastoma, high-grade glioma, and low-grade glioma respectively. The area under the curve (AUC) for each curve; yellow: 0.9525, green: 0.9545, and blue: 0.9901.

**Figure 5:**
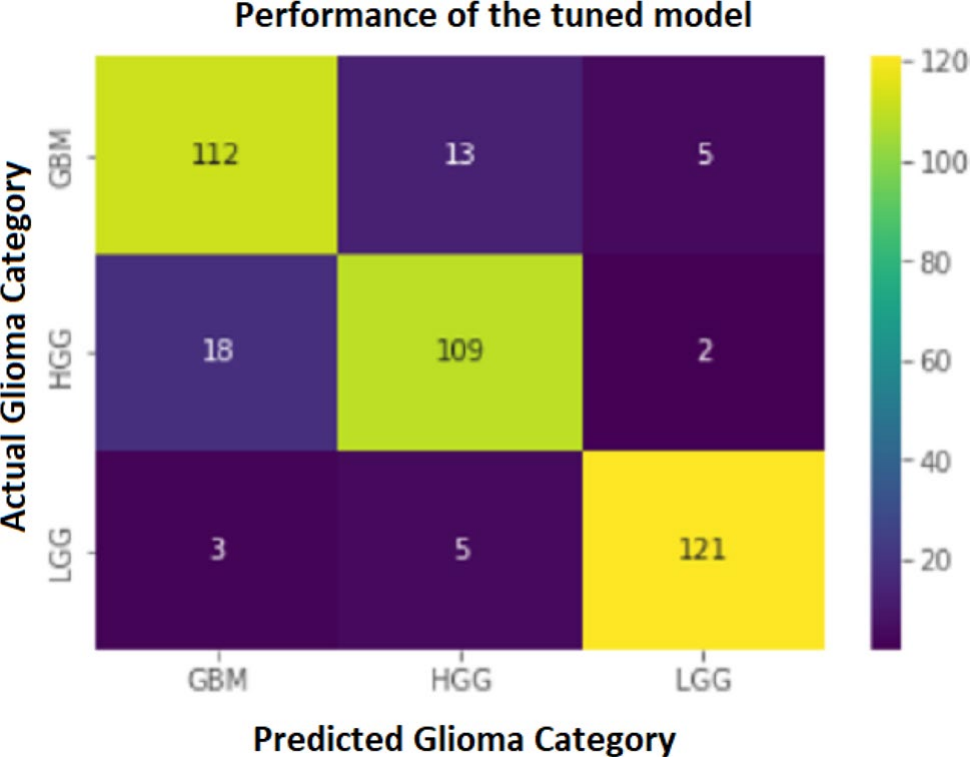
Confusion matrix illustrating the performance of the tuned classification model. According to the confusion matrix, the tuned model predicted 112 out of 130 cases of glioblastoma multiforme (GBM), 109 out of 129 cases of high-grade glioma (HGG), and 121 out of 129 cases of low-grade glioma (LGG).

However, the classification model built by training the Random Forest Classifier algorithm with the train set predicted the glioma categories at 86.08% overall accuracy with a 13.26% average error (see Table 3). According to the area under the curve (AUC) of receiver operating characteristic curve (ROC), the base model performance was glioblastoma vs rest: 0.9434, high-grade glioma vs rest: 0.9521, and low-grade glioma vs rest: 0.9885 (see Fig. 3). After identifying the $min\_samples\_split, n\_estimators, max\_depth, bootstrap, min\_samples\_leaf$, and $max\_features$ as tunable hyperparameters of the base model, the grid search cross-validation technique found the optimum conditions/ combinations of above parameters; $n\_estimators$: 108, *bootstrap*: False, $max\_depth$: 50, $max\_features$: auto, $min\_samples\_leaf$: 1, and $min\_samples\_split$: 2. As a result of assessing the performance of the tuned model using the test set, the tuned model was able to predict the glioma categories with 88.14% accuracy and 11.86% error which is a 2.40% of increment from the accuracy of the base model (see Table  3). Moreover, the tuned classification model correctly predicted 121 out of 129 low-grade glioma image slices, 109 of 129 high-grade gliomas slices, and 112 out of 130 image slices of glioblastomas (see Fig. 5). According to the ROC-AUC values, the tuned model was performed at glioblastoma vs rest: 0.9525, high-grade glioma vs rest: 0.9545, and low-grade glioma vs rest: 0.9901 (see Fig. 4).

**Table 3:** Performance of the developed machine learning model with and without hyperparameter tuning.

	Glioma category	Precision	Recall	f1-score	Support	Accuracy
Base model	0	0.84	0.79	0.81	130	
	1	0.83	0.85	0.84	129	86.08%
	2	0.91	0.94	0.92	129	
Tuned model	0	0.84	0.86	0.85	130	
	1	0.86	0.84	0.85	129	88.14%
	2	0.95	0.94	0.94	129	

The table illustrates the precision, recall, and f1-score acquired by each glioma category in both base model and the tuned classification modes. The glioma categories 0, 1, and 2 represent glioblastoma, high-grade glioma, and low-grade glioma, respectively.

## Discussion

Finding a robust way to identify the severity level or tumor grades of glioma using MRI images has been a leading scientific research area in the past few decades^36^. However, in this study, we discussed about developing an automated and non-invasive method to differentiate gliomas according to the severity level/WHO grades using the information acquired from patient demographics, statistical texture features of GLCM, the mean, skewness, and kurtosis of ADC. However, the intended texture features of each image slice were extracted using homemade software called Brain Lesion Differentiation and Identification Assistant (BLeDIA) which was specifically designed to extract the texture features of MRI brain tumors^29^.

The whole glioma ADC image population acquired from both institutes National Hospital of Sri Lanka (NHSL) and Anuradhapura Teaching Hospital (ATH) was divided into three categories according to the severity of glioma; LGG (WHO I/II), HGG (WHO III), and GBM (WHO IV). According to the statistics, each category contained an unequal number of image slices; 109, 182, and 431 for LGG, HGG, and GBM categories respectively. To avoid the effects of the imbalanced sample sizes of data between each category, the synthetic minority oversampling technique (SMOTE) over-sampling technique was implemented and as a result, the sample sizes of each category were equalized to the sample size of GBM as it has the highest sample size within the population^50,51^.

The results of the cross-validation for seven machine learning classification algorithms indicate that Random Forest Classifier has the highest accuracy score of 0.7901 and Decision Tree Classifier has the second-highest accuracy score of 0.7443 before applying SMOTE. However, after applying SMOTE, the accuracy scores of all the algorithms improved significantly, especially for Gaussian Naïve Bayes, which had the lowest accuracy score before SMOTE application^52,53^. The Random Forest Classifier also had a substantial improvement in accuracy score, with a score of 0.8772, which is the highest accuracy score among all the algorithms after SMOTE application (see Table 2).

Overall, the application of SMOTE technique has positively impacted the performance of all the algorithms, except for Gaussian Naïve Bayes, which had slight decreases in accuracy scores after SMOTE application. the cross-validation results suggest that the application of SMOTE technique can significantly improve the performance of machine learning classification algorithms, especially for imbalanced datasets. However, the impact of SMOTE can vary across different algorithms, and it is essential to evaluate the performance of different algorithms before and after applying SMOTE to determine its effectiveness.

The dataset with equalized sample sizes for each glioma category was split into train and test sets. The most promising algorithm for the data was selected using 10-fold cross-validation. Within this process, the seven most popular supervised learning algorithms were tested and the algorithm that performed the highest cross-validation score with a lesser standard deviation (Random Forest algorithm) was selected to build the classification model (see Table 2).

However, the developed model (base model) could predict the glioma categories with an accuracy of 86.08%, and the high ROC-AUC values calculated in the one versus rest (OVR) method witnessed the high classification power of the developed classifier (see Fig. 4). Also, the performance of the base model over the test set was measured by calculating precision, recall, and f1-score for each glioma category (see Table 3). The accuracy of the base model was optimized by changing the parameters that are critical for the learning process, also known as hyperparameter tuning^54^. At last, the performance of the tuned model was estimated using the test data set and measured by calculating the accuracy and the values of precision, recall, and f1-score for each glioma category. Comparing the precision, recall, and f1-score values of the base model and the tuned model for each category, all the values in the tuned model except the recall score of the HGG category are higher or equal to the precision, recall, and f1-score values of the base model (base model:0.85 > tuned model:0.84) (see Table 3). In addition, the overall classification power of the tuned model for each glioma category was drafted as ROC curves (OVR technique), and the behaviors of the AUC values received for each category in both the base model and the tuned model were compared. As a result, we could identify the improvements in the degree of separability of the tuned model than the base model.

By comparing the results of this study with the study conducted by Alksas et al., in the year 2022, they could reach 95.8% overall prediction accuracy. However, the methodology they used was vastly different from this study. according to their methodology, they have extracted data from several MRI sequences including intravenous (IV) contrast-enhanced sequences such as T1 weighted post-contrast sequence^55^. Young Jin et al., in the year 2014 conducted a study to differentiate gliomas into WHO-II, WHO-III and WHO-IV categories using the features extracted from ADC maps of tumors. They calculated P-*value* for each feature and observed that high-grade gliomas reported significantly higher entropy values and lower fifth percentiles of the ADC cumulative histogram than low-grade tumors. Entropy was the only parameter that was significantly different between grades III and IV, and its diagnostic accuracy was superior to that of the fifth percentile of the ADC histogram in distinguishing high- from low-grade gliomas^56^.

Although the results of this study are promising, there were two main limitations to address when practically executing the study process. The major limitation is drawing the ROIs of 3D tumors in a 2D plane. According to the shape and volume of the tumor, it may appear on several image slices as well as several spots in the same slice. To overcome this problem, we decided to take several ROIs in the same image slice but in different locations and draw ROIs on each image slice that contains the details of the tumor. The next limitation was the lack of patient details. Most of the data collected in this study were accomplished in a retrospective manner. Therefore, tracing the medical records (MRI images, radiological reports, and histopathology reports) of each subject was a challenging event.

## Conclusion

The study concludes that the features extracted and applied in this study such as mean ADC, skewness, kurtosis, GLCM mean 1, GLCM mean 2, GLCM variance 1, GLCM variance 2, entropy, contrast, homogeneity, shade, patients’ age can be collectively used as potential biomarkers to differentiate gliomas according to its severity. Moreover, due to the high accuracy level and the high AUC values of the developed classification model, it can be implemented in clinical setup with further advancements as assistance for clinicians who are involved in the tumor diagnosis process.

## Methods

This prospective study was designed to address the above objective which is building a robust ML model to predict the severity of glioma using the texture features and higher-order moments of MRI-ADC and the patients’ demographics. According to the nature of the collected data and the concerned problem of the study, it was designed as a multi-class classification study and Fig. 1 illustrates the workflow of the supervised learning method utilized in the development of the glioma classification ML model.

### Data acquisition and preparation

The study was carried out using 722 labeled (431 for glioblastoma (GBM)-WHO IV, 182 for high-grade glioma (HGG)-WHO III, and 109 for low-grade glioma (LGG)-WHO I and II) MRI-ADC image slices of 88 human subjects being 57 males, and 31 females who were within the 8 to 90 age range. The pathological condition of each subject was confirmed using the radiological and histopathological reports provided by the experts. All the MRI-DW Digital Imaging and Communications in Medicine (DICOM) data, radiological reports, and corresponding histopathological reports were collaboratively obtained from the departments of Radiology and Histopathology at National Hospital Sri Lanka (NHSL) and the Teaching Hospital Anuradhapura (THA) after obtaining informed consent of the patients, and the ethical clearance approvals from the ethical review board of the Faculty of Medicine, University of Peradeniya, Sri Lanka and the ethical review board of the NHSL. All the data collection activities were carried out within a one-year period under the supervision of the consultants/experts of each institute and department. However, patients with insufficiently detailed or potentially inaccurate information, and damaged/artifact-affected MR images were excluded during the data preprocessing phase.

All the brain tumors that occur without the involvement of glial cells; Meningioma, metastasis, dermoid or epidermoid cysts, choristomas, chondrosarcoma, hamartoma, chordoma, etc., the tumors outside the interested region (extracranial tumors), patients with weak radiological or histopathological histories and corrupted MRI images of brain tumors were excluded at the data preprocessing stage. According to the objectives of this study, the patients’ demographics (age and gender) the mean, skewness (3rd order statistics), kurtosis (4th order statistics) of ADC, and the statistical texture features of GLCM (mean, variance, energy, entropy, contrast, homogeneity, correlation, prominence, and shade) were extracted from the selected subjects.

### Generate ADC images

All the MRI-DW images of the selected subjects were acquired using 3T MR systems and head coils. The Echo Planner Imaging (EPI) sequence with the parameters; TR $= 4300$ ms, TE $= 68$ ms (being TR the time of repetition and TE the time of echo), flip angle = $90^\circ$, field of view (FOV) = $219 \textrm{mm} \times 219 \textrm{mm}$, matrix size $= 124 \times 124$ and slice thickness $= 1$ mm were utilized to generate the required $b = 0 \mathrm{s/mm}^2$, and $b = 1000 \mathrm{s/mm}^2$ DWI images. The DW images generated in two different diffusion sensitization levels *(b-values*); $b = 0\, \mathrm{s/mm}^2$ image, and its corresponding $b = 1000\, \mathrm{s/mm}^2$ image of each patient were collected and utilized to generate ADC images by merging them according to Eq.  (1).

1 $$\begin{aligned} ADC={\sum _{i=1}^{n}} \dfrac{\ln \dfrac{S_i}{S_0}}{b_i}. \end{aligned}$$

Where i represents the image number while the S$_i$ represents the ith image (the image acquired with a diffusion pulse of i). S$_0$ is the first image (image acquired without any diffusion pulses) and n is the number of images and b$_i$ is the diffusion gradient value.

### Region of interest (ROI) selection and feature extraction

**Figure 6:**
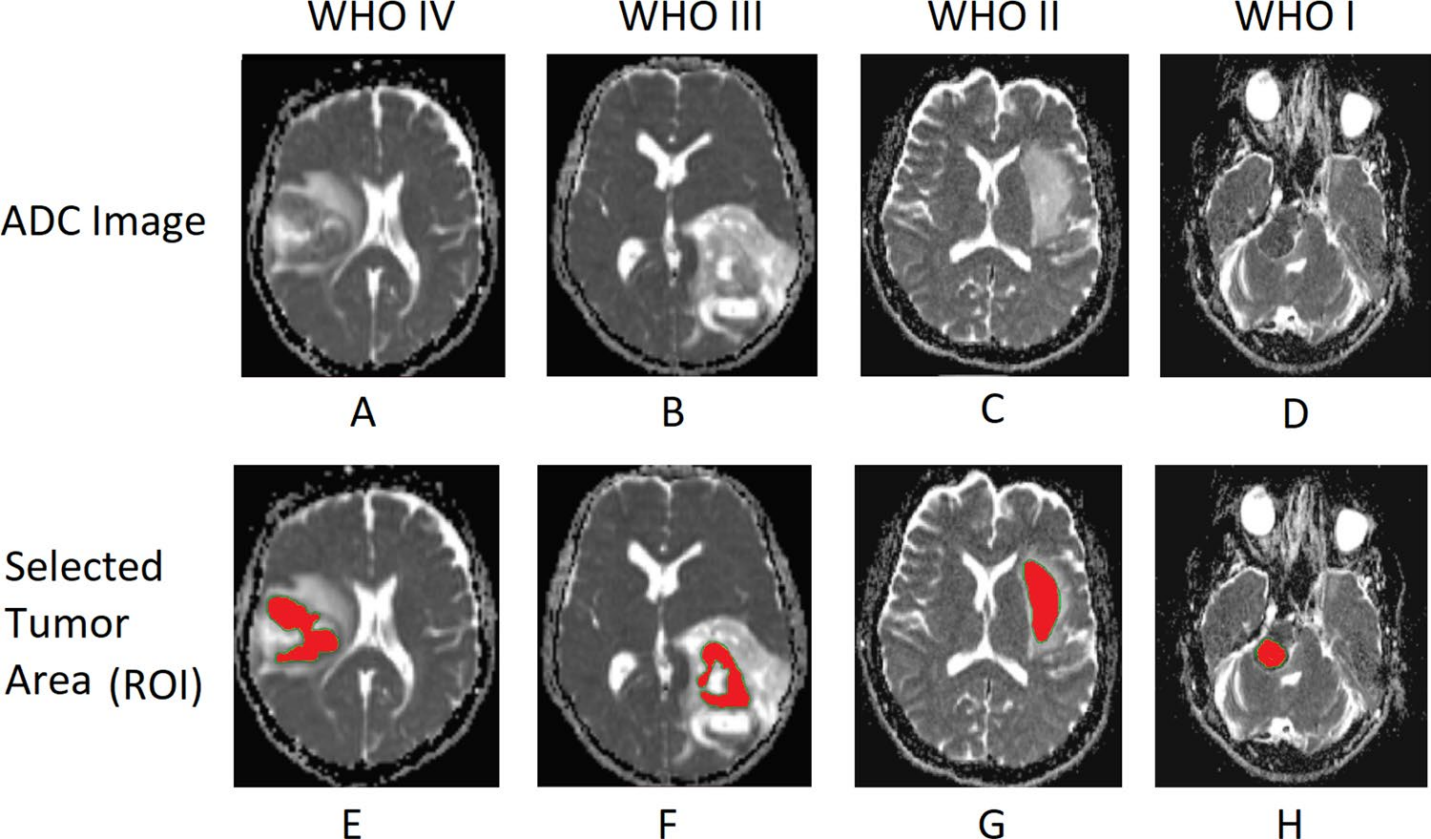
Apparent diffusion coefficient (ADC) images of gliomas. (**A**) An ADC brain image of a 62-year-old male patient presented with glioblastoma multiforme (GBM) (WHO grade IV). (**B**) ADC brain image of a 16-year-old male patient with Anaplastic oligodendroglioma (WHO III). (**C**) ADC brain image of a 39-years-old female patient presented with low grade (WHO II) glioma. (**D**) ADC brain image of a 49-years-old male patient with presented with a schwannoma (WHO I) (**E–H**) illustrate the region of interest drawn over the tumor areas of (**A–D**) images respectively.

the tumor areas within the generated apparent diffusion coefficient (ADC) images of each patient were identified with the assistance of two board-certified consultant radiologists who possess extensive experience in the field of diagnostic radiology, with over 20 years of professional practice. The selection of ADC values within the tumor regions was carried out through the manual drawing of regions of interest (ROI) that encompassed predetermined tumor locations, as illustrated in Fig. 6. All ROIs were delineated manually by a radiology postgraduate student, who was under the strict supervision of the same two consultant radiologists. However, the mean ADC, the higher-order moments of ADC; skewness (3rd order statistics), and kurtosis (4th order statistics) values within the selected ROI were extracted according to Eqs. (2) and (3) respectively. All image processing, ROI selection, and feature extraction processes involved in this study were conducted using custom-made software named Brain Lesion Differentiation and Identification Assistant (BLeDIA) which was developed in Python 3.7.

2 $$\begin{aligned} Mean\_ADC = \dfrac{\sum _{i=1}P_i}{N} \end{aligned}$$

Where p$_i$ is the signal intensity in ith pixel and N is the total number of pixels within the ROI

3 $$\begin{aligned} n^{th}\ moment = \sum _i (P_i - P)^n f(P_i) \end{aligned}$$

Where p$_i$ represents the signal intensity in ith pixel, i represents the number of pixels within the ROI, p represents the mean of the pixel values and f(p$_i$) is the probability of the signal intensity of the pixel.

Using the same ROIs, the GLCM matrices corresponding to each tumor image were generated according to Eq.  (4). The generated GLCM matrices were utilized to extract the statistical texture features of GLCM; mean, variance, energy, entropy, contrast, homogeneity, correlation, prominence, and shade, corresponding to each tumor image. However, most of the texture features are calculated as weighted averages of the normalized GLCM cell contents. Equations  (5) to  (13) describe the methods utilized to extract the statistical texture features of GLCM and within the equations, P$_{i,j}$ represents the probabilities calculated for values in the GLCM matrix, N is the grey levels count in the image, $\mu$ is the mean of P$_{i,j}$ matrix, $\mu _i$ be the mean of row i, $\mu _j$ be the mean value of column j, $\sigma _i$ be the standard deviation of row i and $\sigma _j$ be the standard deviation of column j. The extracted feature values were stored in a CSV file for data preparation and further analysis.

GLCM represents the joint probability occurrence of the pixel pairs containing x and y grey level values for $\delta _\nu$ and $\delta _s$ specific spatial offset between the pixel pairs, and *I* represents the 2D parametric ADC map with dimensions of n $\times$ n (number of grey levels). s and $\nu$ are the spatial positions in image *I* (see Eq.  4).

4 $$\begin{aligned} L_{\delta \nu \delta s}(x,y) = \sum _{x,y=1}^n {\left\{ \begin{array}{ll} 1, \quad \text { if } I(\nu ,s)= x \text { and } I(s+\delta _{s,\nu }+\delta _{\nu }) = y\\ 0, \quad \text {Otherwise} \end{array}\right. } \end{aligned}$$

GLCM Mean: The equation calculates the mean values based on the pixel values of adjacent pixels. in the equation, the left-sided equation calculates the mean based on the pixel with i value ($\mu _{i}$), meanwhile the right-side equation calculates the GLCM mean based on the pixel with j value ($\mu _j$). According to the equation, the similar values for $\mu _i$ and $\mu _j$ indicates that the GLCM matrix is identically symmetrical.

5 $$\begin{aligned} \mu _{i}= \sum _{i,j=0}^{N-1}i\left( P_{i,j} \right) \hspace{2.5cm} \mu _{j}= \sum _{i,j=0}^{N-1}j\left( P_{i,j} \right) \end{aligned}$$

GLCM Variance: GLCM variance measures the dispersion of cell values around the mean. The magnitude of the variance depends on the mean cell values and the dispersion around the mean cell value within the GLCM. Since the GLCM variance is calculated using the GLCM, there is always an involvement of two pixels (the reference (i) and the adjacent (j) pixel). GLCM variance gives the same values for both variances calculated based on pixels value i or j when the matrices are symmetrical

6 $$\begin{aligned} \sigma _{i}^{2}=\sum _{i,j=0}^{N-1}P_{i,j}\left( i-\mu _{i} \right) ^{2} \hspace{2cm} \sigma _{j}^{2}=\sum _{i,j=0}^{N-1}P_{i,j}\left( j-\mu _{j} \right) ^{2} \end{aligned}$$

GLCM Energy (ENR): The GLCM energy measures the uniformity of the grey level distribution of an image. An identically uniform distribution of grey levels in an image (window is very orderly) expresses 1 for GLCM energy and it becomes 0 for images that have an identically nonuniform distribution of grey levels. Here, GLCM energy uses each P$_{(i,j)}$ value as a weight for itself in the calculation of GLCM energy.

7 $$\begin{aligned} ENR=\sum _{i,j=0}^{N-1}P_{i,j}^{2} \end{aligned}$$

GLCM entropy (ENT): Describes the degree of disorder among pixels within the matrix, which is approximately inversely correlated with uniformity. The Larger the number of grey levels within the image expresses larger entropy values.

8 $$\begin{aligned} ENT = \sum _{i,j=0}^{N-1}P_{i,j}\left( -\ln P_{i,j} \right) \end{aligned}$$

GLCM contrast (CNT): GLCM contrast, also known as the sum of squares variance, measures the intensity difference between two neighboring pixels (i, and j) over the whole image. GLCM contrast becomes 0 for constant images (i-j), while the weights continue to increase exponentially as the difference of pixel intensities (i-j) increases. However, the edges, noise, or wrinkled textures within an image increase the contrast value.

9 $$\begin{aligned} CTN =\sum _{i,j=0}^{N-1}P_{i,j}\left( i-j \right) ^2 \end{aligned}$$

GLCM homogeneity (HOM): GLCM Homogeneity is the way of measuring the smoothness of distribution of gray levels within an image, which is inversely correlated with contrast.

10 $$\begin{aligned} HOM = \sum _{i,j=0}^{N-1}\frac{P_{i,j}}{1+\left( i-j \right) ^{2}} \end{aligned}$$

GLCM correlation (COR): The linear dependency of grey levels on neighboring pixels of the image is measured by the GLCM correlation. When there is a linear and predictable relationship between the two pixels, the corresponding correlation increases. Therefore, the images with high correlation values express that there is high predictability of pixel relationship.

11 $$\begin{aligned} COR = \sum _{i,j=0}^{N-1}P_{i,j}\left[ \frac{\left( i-\mu _{i} \right) \left( j-\mu _{j} \right) }{\sqrt{\left( \sigma _{i}^{2} \right) \left( \sigma _{j}^{2} \right) }} \right] \end{aligned}$$

GLCM cluster shade (CS): Evaluate the tendency of clustering of the pixels by measuring the skewness of pixel values within the matrix. GLCM Cluster shade measures the uniformity of a grey image and values fluctuate between 0 to 2. Therefore, the higher values for cluster shade indicate the nonuniform distribution of grey values in the image.

12 $$\begin{aligned} CS = \sum _{i,j=0}^{N-1}\left\{ i+j-\mu _{i}-\mu _{j} \right\} ^{3}P_{i,j} \end{aligned}$$

GLCM cluster prominence (CP): Measures local intensity variation of pixels and the asymmetry of an image. A high prominence value indicates less symmetry of an image while an image with a less cluster prominence value shows the peak in the GLCM matrix around the mean.

13 $$\begin{aligned} CP = \sum _{i,j=0}^{N-1}\left\{ i+j-\mu _{i}-\mu _{j} \right\} ^{4}P_{i,j} \end{aligned}$$

### Feature selection and model training

Following the extraction of GLCM texture features, mean ADC, and the higher-order moments of ADC, the demographic data corresponding to each subject was taken to a single spreadsheet. Then, all the feature values corresponding to each image slice were labeled manually with the final diagnosis. According to the labels, the dataset was divided into three classes; Glioblastoma (WHO IV), High-grade glioma (WHO III), and Low-grade glioma (WHO I and II). However, the sample size of each class was not equal to each other.

Therefore, the Synthetic Minority Over-sampling Technique (SMOTE) was utilized to balance the imbalanced sample sizes of each class. SMOTE generates synthetic examples of the minority class by following a set of steps. Initially, a random minority class example is selected, and its k-nearest neighbors from the same minority class are identified. Then, one of the k-nearest neighbors is randomly chosen. A new synthetic example is produced by interpolating between the selected example and the randomly selected nearest neighbor. The interpolation involves computing a weighted sum of the feature values of the two examples. The process continues until the required number of synthetic examples has been generated. The outcome of this algorithm is an increase in the number of minority class examples, which can enhance the performance of classifiers that are biased toward the majority class.

Data within each class of imbalanced (before applying SMOTE) and balanced (after applying SMOTE) datasets were split into train and test sets with a proportion of 70%:30%, respectively by keeping the random state at 42. The purpose of considering the states of the dataset before and after applying SMOTE was to evaluate the effect of SMOTE in developing ML models. Then the features in each train set were standardized as all the features centered around zero mean and unit variance. This standardization process avoids the domination of features with high variance in the learning process. Therefore, it leads the estimator to learn from other features correctly and unbiasedly (see Eq.  14).

14 $$\begin{aligned} A_{n}= \frac{A-A_{\min }}{A_{\max }-A_{\min }}. \end{aligned}$$

Where $A_{n}$ is the normalized value of a feature value, *A* is the feature value,$A_{\max }$ and $A_{\min }$ represents the maximum and minimum values reported for the considering feature

Among the standardized features in both the balanced and balanced datasets, the subset of input features that are most relevant to the target variables (classes) was selected using the ANOVA (Analysis of Variance) f-test feature selection method. Specifically, the entire training dataset was subjected to the ANOVA f-test feature selection algorithm, and the three features that performed minimum scores on the test (i.e., features that are primarily independent of the target variable) were excluded from each dataset (see Fig.  2). The remaining features were then used in the subsequent K-fold cross-validation experiment to identify the most promising machine learning (ML) algorithm for each dataset.

To this end, a tenfold (K $=$ 10) cross-validation experiment was conducted over both training datasets using several common classification algorithms, including Logistic Regression, Linear Discriminant Analysis, Decision Tree Classifier, Gaussian Naïve Bayes, Support Vector Machine (SVM), K-nearest neighbor (KNN), and Random Forest Classifier. The ML algorithm that yielded the highest cross-validation score was considered as the most promising algorithm to develop the glioma classification model. However, the impact of the application of SMOTE oversampling technique was also examined by comparing the results of the 10-fold cross-validation of both datasets. Here, Python 3.7 along with the scikit-learn library was utilized to standardize the data, ANOVA F-test, SMOTE oversampling, and building and assessing the classification models.

**Table 4:** Default parameters used to build the base model.

Parameter	Status
Bootstrap	True
ccp_alpha	0.0
class_weight	None
Criterion	Gini
max_depth	None
max_features	Auto
max_leaf_nodes	None
max_samples	None
min_samples_leaf	1
min_samples_split	2
min_weight_fraction_leaf	0.0
n_estimators	100
n_jobs	None
oob_score	False
random_state	None
Verbose	0
warm_start	False

The base model was developed using the illustrated default parameters and conditions.

According to the K-fold cross-validation experiment, the Random Forest Classifier was selected as the most promising algorithm to build the glioma grading classification model. The Random Forest Classifier algorithm was trained with the train data by keeping the random state at 42, and the developed classification model (base model) was evaluated using the test set. This developed base model consisted of default parameters (see Table  4), and the performance of the base model was assessed by using the overall accuracy, precision, recall, and F1 scores corresponding to each class. However, the combination of tunable hyperparameters; min_samples_split, n_estimators, max_depth, bootstrap, min_samples_leaf, and max_features, of the developed model was optimized (tuned) using the grid search cross-validation technique on the training set, in order to improve the performance of the model. Here, each hyperparameter was tested within a pre-defined range of values; n_estimators: from 100 to 1000 (with the step of 400), min_samples_split: (2, 5, 10), max_depth: 10 to 110 (with 11 steps), max_features: auto, and bootstrap: True, False. The tuned model was also evaluated using the test set and the performance of the tuned model was assessed by observing the overall accuracy, precision, recall, and the F1 scores. Also, the performance of the developed model was graphically illustrated using receiver operating characteristic (ROC) curves for each class using the one-vs-rest (OVR) technique and quantitatively measured the performance by calculating the area under the curve (AUC) (see Figs. 3  and  4).

### Ethics approval and consent to participate

The study was approved by two institutional ethics review committees and All the methods were carried out in accordance with relevant guidelines and regulations. (1) The ethics review committee of the Faculty of Medicine, University of Peradeniya under 2019/EC/50 reference number. (2) The ethics review committee of the National Hospital of Sri Lanka, Colombo 10, under ETH/COM/2019/AUGUST/05 reference number.

## Data Availability

The data that support the findings of this study are available on request from the corresponding author [M. L. Jayatilake]. The data are not publicly available due to them containing information that could compromise research participant privacy, and consent.

## Abbreviations

MRI: Magnetic resonance imaging
DW: Diffusion weighted
DWI: Diffusion weighted imaging
CNS: Central nervous system
GBM: Glioblastoma multiforme
LGG: Low grade glioma
HGG: High grade glioma
ADC: Apparent diffusion coefficient
GLCM: Grey level co-occurrence matrix
ML: Machine learning
ROC: Receiver operating characteristic curve
